# Motor Imagery Combined With Physical Training Improves Response Inhibition in the Stop Signal Task

**DOI:** 10.3389/fpsyg.2022.905579

**Published:** 2022-06-20

**Authors:** Sung Min Son, Seong Ho Yun, Jung Won Kwon

**Affiliations:** ^1^Department of Physical Therapy, College of Health Science, Cheongju University, Cheongju, South Korea; ^2^Department of Public Health Sciences, Graduate School, Dankook University, Cheonan, South Korea; ^3^Department of Physical Therapy, College of Health and Welfare Sciences, Cheonan, South Korea

**Keywords:** motor imagery, response inhibition, stop signal task, stop signal reaction time, motor imagery with physical training

## Abstract

**Background:**

Motor imagery training has a similar effect to that of physical training on motor performance. The objective of this study was to investigate the short-term effectiveness of motor imagery training on response inhibition using the stop signal task (SST).

**Methods:**

Participants were divided into a physical training group (PT, *n* = 17), a motor imagery training group (MIT, *n* = 17), and a motor imagery combined with physical training group (MIPT, *n* = 17). All participants performed 10 SST training sessions over 5 days. Both stop signal reaction time (SSRT) and non-signal reaction time (NSRT) were measured before and after SST training.

**Results:**

There were significant interaction (time × group) and time effects, although the group effect was not statistically significant. Bonferroni *post hoc* analysis showed that MIPT group revealed a significantly greater change in SSRT than PT and MIT groups, while there was no significant difference between PT and MIT groups. SSRT significantly decreased after training in all groups. In NSRT, there was a significant effect of time, but there was no significant interaction effect (time × group) or group effect.

**Conclusion:**

Response inhibition could be enhanced *via* training, and it was most effective when motor imagery and physical training were combined. We demonstrate that motor imagery training significantly improves response inhibition and should be accompanied by physical training when performing SST.

## Introduction

Response inhibition is a complex cognitive process which allows suppression of preponent and inappropriate responses (Miyake et al., 2000; Aron, 2007). It is required for goal-directed behaviors in a changing environment (Barkley, 1997). The ability to perform response inhibition is usually evaluated using the stop-signal task (SST), which is based on the stop-signal paradigm, proposed by Logan for the study of response inhibition (Logan, 1994). The SST consists of two trials: “go trial” and “stop trial” associated with response execution and inhibition, respectively. It has a primary advantage over other response inhibition control tasks (e.g., Go/No Go and flanker tasks) because it indirectly determines the latency of response inhibition by measuring the stop signal reaction time (SSRT; Bari and Robbins, 2013). The SSRT is a reliable indicator for evaluating inhibitory control ability and proficiency in SST; a shorter SSRT indicates a faster stop process and thus a more efficient response inhibition (Verbruggen and Logan, 2009b). Many studies have reported that SSRT can be used to identify deficits in response inhibition in patients with neurological and psychopathological diseases, such as Parkinson’s disease, attention-deficit/hyperactivity disorder, and obsessive–compulsive disorder (Sea et al., 2000; Chamberlain et al., 2006; Mirabella et al., 2017; Di Caprio et al., 2020). Indeed, they showed longer SSRT when compared to healthy individuals, indicating a impaired response inhibition (Sea et al., 2000, Chamberlain et al., 2006, Mirabella et al., 2017, Di Caprio et al., 2020). Based on these previous studies, SSRT has been useful for comparing response inhibition in diverse conditions.

Response inhibition is associated with the fronto-basal-ganglia network consisting mainly inferior frontal gyrus, pre-supplementary motor area, and basal ganglia (Aron and Poldrack, 2006; Verbruggen and Logan, 2008; Aron, 2011). In addition, the primary motor cortex is related to response inhibition (Chowdhury et al., 2020). Many previous studies have attempted to determine the neural basis of response inhibition (Aron and Poldrack, 2006; Verbruggen and Logan, 2008; Aron, 2011; Chowdhury et al., 2020). The fronto-basal-ganglia network, consisting of the inferior frontal gyrus, pre-supplementary motor area, and basal ganglia, has been identified as crucial for response inhibition (Aron and Poldrack, 2006; Verbruggen and Logan, 2008; Aron, 2011). However, the precise roles of these regions remain debatable. Some previous studies suggested that the right inferior frontal gyrus and pre-supplementary motor area are not exclusively activated by response inhibition but may be part of domain general functional networks that control diverse cognitive processess including attentional capture (Sharp et al., 2010), working memory (Hampshire, 2015; Hampshire and Sharp, 2015), and motor planning and execution (Mirabella, 2014). Additionally, the primary motor cortex, which is the final target of inhibitory command (Mattia et al., 2012), contributes to response inhibition by suppressing descending motor output (Coxon et al., 2006; Stinear et al., 2009; Chowdhury et al., 2020). In a stop signal paradigm, anodal transcranial direct current stimulation to the primary motor area could decrease the time of go and stop trials (Kwon et al., 2013). These findings suggest that increased cortical activity in the primary motor cortex improves go and stop processes.

SST training can improve the ability of response inhibition by altering the neural network (e.g., the fronto-basal-ganglia network; Manuel et al., 2013). An electrical neuroimaging study which investigated the effects of SST training on the fronto-basal-ganglia network and the ability of response inhibition found decreased SSRT after 1 hour of SST training (Manuel et al., 2013). This result was related to decreased activity in the right inferior frontal gyrus, pre-supplementary motor area, and basal ganglia during response inhibition. Thus, SST training can lead to the effectiveness of motor learning that increases the efficiency of neural activity by excluding irrelevant neural activity.

Motor learning is associated with plastic changes in the sensorimotor systems as a result of repeated practice and feedback (Newell, 1991). Although physical training is recognized as the primary approach for motor learning (Kraeutner et al., 2016), motor imagery has been demonstrated as an effective alternate method for facilitating motor learning in diverse disciplines (e.g., sports, cognitive, sport psychology; Gentili et al., 2006, Robin et al., 2007, Frank et al., 2014). Motor imagery is defined as the act of imagining a motor action without the involvement of physical movement, thus allowing the subject to mentally experience movements, even those that are physically impossible (Murphy, 1994). Some brain areas activated during motor imagery are similar to those activated during physical movements under the same conditions (Decety et al., 1988; Stephan et al., 1995; Gerardin et al., 2000). Motor imagery can activate parts of the motor system as well as other cortical and subcortical regions (e.g., basal ganglia and cerebellum) and enhance neural pathways associated with physical movement (Decety et al., 1994; Gerardin et al., 2000, Lui et al., 2008). In this way, motor imagery training can lead to improved motor performance without involving any physical movement (Landers, 1983; Dickstein and Deutsch, 2007). Many studies have reported that motor imagery training has positive effects on motor performance and produces similar cortical plastic changes in neurologically injured patients or athletes (Lebon et al., 2010; Braun et al., 2013; Cho et al., 2013; El-Wishy and Fayez, 2013; Taube et al., 2015). In particular, motor imagery training has shown to improve gait (Cho et al., 2013; El-Wishy and Fayez, 2013), balance (Cho et al., 2013; Taube et al., 2015), motor planning (Braun et al., 2013), and muscle strength (Lebon et al., 2010). Furthermore, its effectiveness for improving motor performance increases when combined with physical training or action observation compared to motor imagery or physical training performed independently (Feltz and Landers, 1983; Taube et al., 2015).

Based on these findings, motor imagery training can be useful for improving motor performance. However, there is currently a lack of evidence regarding its effects on response inhibition. Furthermore, it is necessary to understand whether and how motor imagery training affects response inhibition when combined with physical training in order to determine the most effective training method. Therefore, the purpose of this study was to investigate the effect of motor imagery training on response inhibition when performed alone and in combination with physical training. We hypothesized that the SSRT would be reduced after motor imagery training and physical training. Its change would be greater when motor imagery training is combined with physical training than when either motor imagery training or physical training is performed alone. The non-stop signal reaction time (NSRT) would not change after physical training and motor imagery training.

## Materials and Methods

### Participants

The sample size was calculated using the G-power software (G*power 3.1.9.7, Heinrich-Heine-Universität, Düsseldorf, Germany). With an effect size of 0.25, a significance level of 0.05, and a power of 0.80, a minimum of 42 participants was adequate to power the study. This study included 51 undergraduate students with no history of musculoskeletal, neurological, or psychiatric diseases. Participants were recruited through posters and an electronic research bulletin board on campus. They had not previously participated in any sequence-learning experiments that could have affected the SST. Participants were randomly assigned to one of three groups according to how SST training was performed: physical training, motor imagery training, or motor imagery combined with physical training. All participants provided written informed consent in accordance with the Declaration of Helsinki. This study was approved by the Institutional Review Board of Dankook University (DKU 2021-03-062).

### Stop Signal Task

The STOP-IT program (Universiteit Ghent, Belgium) was used to perform the SST. The SST consists of go and stop trials, which account for 75% and 25% of the trials, respectively. A fixation cross was presented at the center of the black screen until the participants respond, or until 1,250 msec have passed. Each trial began with the presentation of the fixation cross, which was replaced by a go signal after 250 msec. In go trials, a go signal, such as a square (■) or a circle (●), was randomly presented as a visual stimulus at the center of the monitor. In stop trials, the stop signal, in the shape of an X, was presented following a variable delay (the stop-signal delay; SSD) after the go signal. Go and stop signals remained in the computer monitor until the participants reacted or for 1,250 msec in case there was no reaction. A trial was excluded from analysis if a button was pressed prior to the presentation of the go signal, and if the incorrect button was pressed in response to the signal.

The SSD refers to the amount of time between the go and stop signals. The initial SSD was 250 msec; it was increased by 50 msec with a successful stop trial and decreased by 50 msec with a failed stop trial (Verbruggen et al., 2008). In this way, SSD was continuously adjusted with the tracking procedure to obtain a probability of successfully stopping at 50% (Clark et al., 2020). The indicators for the extent of the response inhibition include the SSRT, which represents the latency of the response time in stop trials, and the NSRT, which represents the response time for the go trials. SSRT was calculated by subtracting the mean SSD from the mean NSRT (SSRT = mean NSRT—mean SSD; Band et al., 2003, Verbruggen and Logan, 2009b). The analysis of the quantified SSRT and NSRT was carried out using the STOP-IT analysis program (ANALYZE-IT, Universiteit Gent, Belgium).

### Procedure

This study employed a three-group, pre-test–post-test design. The three groups were defined as physical training (PT, *n* = 17), motor imagery training (MIT, *n* = 17), and motor imagery combined with physical training group (MIPT, *n* = 17). The subjects were comfortably seated in a chair at 70 cm from the front of the monitor where the signal was presented. The subjects first performed a pre-SST test, which consisted of an adaptation phase of 32 trials (i.e., 24 go and 8 stop trials) and a test phase of 192 trials (i.e., 144 go and 48 stop trials) for the acquisition of baseline parameters (pre-SSRT and pre-NSRT). In the go trials, the subjects were asked to press the button using their non-dominant hand according to the corresponding signal as quickly as possible. The go signals were as follows: squares (■) were associated with left-facing arrows (←), an indication for pressing a specific button located on the left of the keyboard; whereas circles (●), associated with right-facing arrows (→), meant that a button located at a certain place on the right of the keyboard should be pressed. In the stop trials, the subjects were asked not to press any button in response to the stop signal, which presented the shape of an X and appeared at variable times (SSD) once the go signal was already initiated.

A day following the pre-test, SST training using the same task as the pre-test was performed with two types: motor imagery training and physical training sessions. In the motor imagery training session, subjects were asked to imagine the response to the go and stop stimuli without pressing the button, while watching the recorded pre-SST test video. In the physical training session, subjects were asked to practice SST by pressing a button. Each of motor imagery training and physical training sessions consisted of 224 trials (i.e., 168 go trials and 56 stop trials). In one SST training, the MIPT group performed one motor imagery training (224 trials) and one physical training (224 trials) sessions, while the MIT group performed twice motor imagery training sessions (448 trials), and the PT group conducted twice physical training sessions (448 trials). Thus, all groups completed a total of 448 trials during one SST training. All participants performed the SST training five times over 5 days. All groups performed the post-SST test in the same manner as the pre-SST test the day after the training ended.

### Data Analysis

Data analysis was performed using JASP (Version 0.16.2, Amsterdam, The Netherlands). The Shapiro–Wilk test was used for normality testing among the measurements. Baseline differences were compared using one-way ANOVA (age) and chi-square test (sex). 3 × 2 (group × time) mixed ANOVA with time as a within factor and group as a between factor was used to assess the effects of the time and group, as well as their interaction on the probability of failure, SSRT and NSRT. For *post hoc* analysis, Bonferroni adjustment was used. The use of only value of *p* for statistical analysis has been recently widely criticized; frequentist analyses do not allow gathering evidence for accepting or rejecting the null hypothesis (Wetzels et al., 2011; Wagenmakers et al., 2016). This frequentist analyses problem can be supplemented by Bayesian analyses because Bayesian analyses do not assume large samples, and typically smaller sizes can be analyzed without losing power while retaining precision (Lee and Song, 2004; Hox et al., 2012). We employed the Bayesian repeated measure ANOVA with factor time (pre- and post-test) and group (PT, MIT, and MIPT) to extend the explanatory power of the frequentist interference. BF_10_ is a ratio of evidence supporting the alternative hypothesis relative to the null hypothesis (Lee and Wagenmakers, 2013). For interpretation of the BF10, the following classifications related to the strength of the evidence for the alternative hypothesis relative to the null hypothesis were used: no evidence (BF10 = 1), anecdotal evidence (1 < BF10 ≤ 3), substantial evidence (3 < BF10 ≤ 10), strong evidence (10 < BF10 ≤ 30), very strong evidence (30 < BF10 ≤ 100), or decisive evidence (BF10 > 100; Lee and Wagenmakers, 2013). A statistically significant test result (*p* ≤ 0.05) means that the test hypothesis is false or should be rejected.

## Results

Table 1 presents the participants’ demographic data. There were no significant differences in sex or age among the groups (*p* > 0.05).

**Table 1:** General characteristics of each group.

	PT group	MIT group	MIPT group	*p*
Gender				
M	10	9	9	0.924
F	7	8	8	
Age (yr)	22.82 ± 2.63	22.59 ± 1.84	22.76 ± 1.79	0.945

Mean ± SD. PT, physical training; MIT, motor imagery training; and MIPT, motor imagery with physical training.

**Figure 1:**
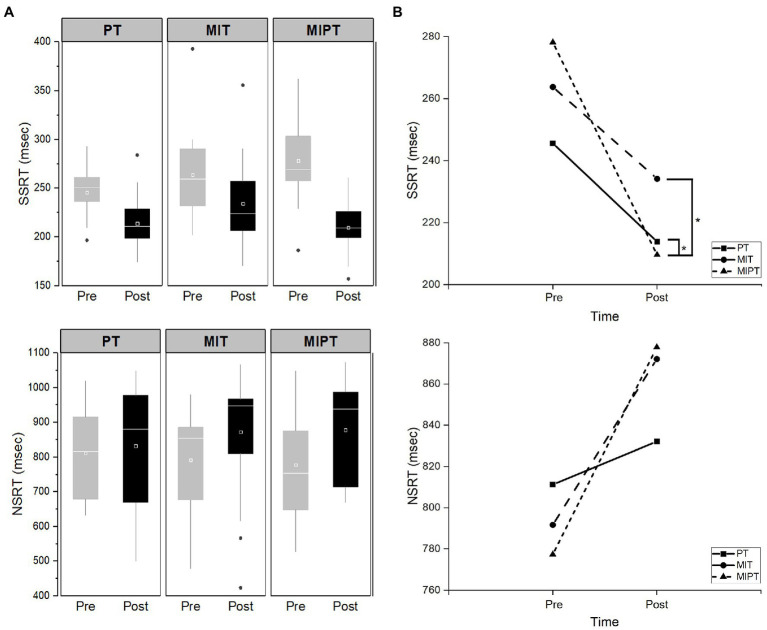
Stop signal reaction time (SSRT) and non-signal reaction time (NSRT) values between pre- and post-test in each group. **(A)** SSRT and NSRT values between pre- and post-test in each group, **(B)** the *post hoc* analysis in SSRT and NSRT. Data are presented as mean values ± SD. PT, physical training; MIT, motor imagery training; and MIPT, motor imagery with physical training. ^*^Indicates statistical differences as confirmed by Bonferroni *post hoc* test (*p* < 0.05).

Table 2 shows the behavior values for the three groups during the SST. First, we assessed how the staircase algorithm worked in all groups. There were no significant effects of time [F (1,28) = 2.872, *η_p_*^2^ = 0.093, *p* = 0.101], group [F (2,28) = 0.205, *η_p_*^2^ = 0.014, *p* = 0.816], and interaction (time × group)[F (2,28) = 1.326, *η_p_*^2^ = 0.086, *p* = 0.282] on the probability of failure (*p* > 0.05). In addition, the validity of the stochastic independence between go and stop processes was evaluated *via* the mixed-design ANOVA with trial type (reaction time of no-stop trials, reaction time of stop-failure trials) as a within factor and group (PT, MIT, and MIPT) as a between factor. The mixed design ANOVA showed that stop-failure trials were faster than no-stop trials [F (1,228) = 626.31, *η_p_*^2^ = 0.733 *p* < 0.001], which indicated that all groups fulfilled the assumption of the race model (Logan et al., 1984). There was no significant difference in the pre-SSRT scores among the groups (*p* > 0.05). The mixed-design ANOVA on SSRT revealed a significant main effect of time [F (1,48) = 56.78, *η_p_*^2^ = 0.542, *p* < 0.001] and significant interaction between time and group [F (2,48) = 4.84, *η_p_*^2^ = 0.168, *p* = 0.012], but the group effect was not statistically significant [F (2,48) = 1.724, *η_p_*^2^ = 0.067, *p* = 0.189; Table 2]. This frequentist results were supported by the Bayesian analysis. The Bayesian model comparisons supported models that included time (BF_10_ = 7.850 × 10^6^) and time × group (BF_10_ = 2.562 × 10^7^) when compared to the null model. *Post hoc* analysis showed that MIPT group evidenced a significantly greater change in SSRT compared to both PT and MIT groups (*p* = 0.031, *p* = 0.022), although there was no significant difference between PT and MIT groups (*p* = 0.98; Figure 1). The SSRT significantly decreased after training in all groups (*p* < 0.05; Appendix 1).

**Table 2:** Behavioral values for the three groups during the stop-signal task.

	PT group	MIT group	MIPT group	Time	Group	Time × group
SSRT						
Pre	245.60 ± 28.47	263.76 ± 46.19	278.13 ± 41.55	*F* = 56.784 *η_p_*^2^ = 0.542 *p* = < 0.001^*^ BF_10_ = 7.850 × 10^6^	*F* = 1.724 *η_p_*^2^ = 0.067 *p* = 0.189 BF_10_ = 0.351	*F* = 4.835 *η_p_*^2^ = 0.168 *p* = 0.012^*^ BF_10_ = 2.562 × 10^7^
Post	213.88 ± 29.21	234.14 ± 44.63	209.61 ± 29.52			
Difference	−31.72 ± 29.66	−29.62 ± 48.66	−68.52 ± 42.44			
NSRT						
Pre	811.25 ± 128.81	791.73 ± 146.79	777.35 ± 145.70	*F* = 11.198 *η_p_*^2^ = 0.189 *p* = 0.002^*^ BF_10_ = 17.926	*F* = 0.024 *η_p_*^2^ = 0.001 *p* = 0.977 BF_10_ = 0.193	*F* = 1.414 *η_p_*^2^ = 0.056 *p* = 0.253 BF_10_ = 1.571
Post	832.14 ± 179.42	872.14 ± 178.74	877.86 ± 147.36			
Difference	20.88 ± 148.24	80.41 ± 116.85	100.511 ± 161.85			
*p* (r|s)						
Pre	44.65 ± 2.50	45.46 ± 3.67	45.33 ± 4.34	*F* = 2.872 *η_p_*^2^ = 0.093 *p* = 0.101 BF_10_ = 1.132	*F* = 0.205 *η_p_*^2^ = 0.014 *p* = 0.816 BF_10_ = 0.224	*F* = 1.326 *η_p_*^2^ = 0.086 *p* = 0.282 BF_10_ = 0.142
Post	47.79 ± 4.40	45.36 ± 2.44	46.44 ± 3.71			

Mean ± SD. PT, physical training; MIT, motor imagery training; and MIPT, motor imagery with physical training.

* *p* < 0.05.

Regarding NSRT, there was no significant difference in pre-NSRT scores among the groups (*p* > 0.05). The mixed-design ANOVA on NSRT revealed a significant main effect of time [F (1,48) = 11.20, *η_p_*^2^ = 0.189, *p* = 0.002], although there were no significant effects of either interaction between time and group [*F* (2,48) = 1.41, *η_p_*^2^ = 0.056, *p* = 0.253] and group [F (2,48) = 0.024, *η_p_*^2^ = 0.001, *p* = 0.977; Table 2]. The Bayesian model comparisons supported models that included time when compared to the null model (BF_10_ = 17.926). The NSRT significantly increased after training in both MIT and MIPT groups (*p* = 0.0025, *p* = 0.006); however, there was no significant difference in PT group (*p* = 0.551).

## Discussion

We investigated the effects of motor imagery training on response inhibition when performed alone and in combination with physical training. To the best of our knowledge, this is the first study to examine the changes in response inhibition after motor imagery training. The main findings of this study were as follows: (1), SSRT after physical training was significantly decreased in the PT group; (2), SSRT after motor imagery training was significantly decreased in the MIT group; (3), SSRT after motor imagery training with physical training was significantly decreased in the MIPT group; and (4), changes in SSRT were significantly greater in the MIPT group than in the MIT and PT groups. These results indicate that response inhibition can be enhanced *via* training, and that training was most effective when motor imagery training and physical training were combined.

In the present study, SSRT significantly decreased in the PT group. Our findings are consistent with those from previous studies which demonstrated that physical training of SST leads to improvement in response inhibition, although the training periods differed between studies. Indeed, previous studies have reported that SSRT decreased after short-term physical training with SST (Kwon et al., 2011; Manuel et al., 2013). This improvement was associated with a decreased internal processing required for response inhibition (Kwon et al., 2011). Many previous studies reported that right-lateralized fronto-basal-ganglia network was core network of response inhibition (Aron and Poldrack, 2006; Verbruggen and Logan, 2008; Aron, 2011). However, the exact role of these structures is unclear and debated. Wheras some researches have argued that the right inferior frontal gyus is a crucial node within the right-lateralized fronto-basal-ganglia network for response inhibition (Aron et al., 2014, 2015), others have suggested that this region is only one out an ensemble of right prefrontal regions, which implement inhibitory control (Hampshire, 2015; Hampshire and Sharp, 2015). The right inferior frontal gyrus and pre-supplementary motor area are components of domain general networks that control cognitive processes such as attentional control (Sharp et al., 2010), working memory (Hampshire, 2015, Hampshire and Sharp, 2015), action planning and execution (Mirabella, 2014). Successful response inhibition occurs from dynamic interaction throughout these networks (Leunissen et al., 2016). The activity of the right inferior frontal gyrus, pre-supplementary motor area, primary motor cortex and basal ganglia were changed after physical training of SST. A decrease in activity in these areas imply that physical training with SST may increase the efficiency of neurial activity by excluding irrelevant neural activity and increasing the selectivity (Kelly and Garavan, 2005). Together with these studies, our results indicate that physical training with SST is effective for motor learning of response inhibition.

We also found that SSRT significantly decreased in MIT and MIPT groups. Motor imagery is a complementary method for motor learning that also continuously induces neural plasticity and reorganization of the involved brain regions and circuits (Schuster et al., 2011). The ability of a neural circuit to reorganize is an important component of motor learning (Munzert et al., 2009). The learning outcome of motor imagery training can be indirectly evaluated as a behavioral or neural outcome because the motor imagery process cannot be directly observed (Munzert et al., 2009). In terms of behavioral outcomes, the SSRT is a reliable index of the ability to respond to inhibition and proficiency in SST (Congdon et al., 2012). Thus, a decreased SSRT in MIT and MIPT groups indicated that their response inhibition improved as a result of motor imagery training. Furthermore, motor imagery training is more effective for motor learning in complex cognitive and motor tasks than in simple ones (Allami et al., 2008). In this sense, the ability of response inhibition is described as a higher-level motor and cognitive function which acts regulating interference control, delaying the prepotent response, and stopping the response in progress (Friedman and Miyake, 2004). In the context of previous studies, our results indicate that motor imagery training improves response inhibition, resulting in motor learning in a complex cognitive function.

In the present study, the change in SSRT was significantly greater in the MIPT group than in the MIT and PT groups. Motor imagery training combined with physical training has proven to be more effective for motor learning than either physical training or motor imagery training performed individually. In this regard, Frank et al. (2014) investigated the most effective training strategies for improving motor performance and mental representation structures in novice. They reported that motor imagery training combined with physical training was more effective than each of these treatments alone in improving motor performance and mental representation. According to the cognitive action architecture approach, motor learning can be achieved through continuous modification and adaptation of the mental representation structure (Schack, 2004; Schack and Mechsner, 2006; Schack and Ritter, 2009), which in turn comprises cognitive elements involved in goal-directed movement in motor learning (Frank et al., 2014). It is suggested that mental-physical combined training promotes a cognitive adaptation process during motor learning by more structured and elaborated mental representations (Frank et al., 2014). Therefore, our results indicate that when motor imagery training is combined with physical training, the cognitive adaptation process is enhanced, resulting in motor learning of response inhibition.

No significant difference was observed regarding the change in the SSRT between the MIT and PT groups. Although motor imagery training shares the neural mechanism of physical movement, controversies exist as to whether motor imagery training is more effective in improving behavioral outcomes than physical training. While it has been argued that motor imagery training has a less positive effect on performance than physical training, (Landers, 1983; Driskell et al., 1994) other studies suggested that the effects of motor imagery training are equal to or greater than those of physical training (Wohldmann et al., 2008). These contradictory results may be due to the differences in the type and purpose of the task. Task type was identified as a factor for which the outcome of motor imagery training may vary, with greater effects reported in cognitive tasks compared to physical tasks (Ladda et al., 2021). Although a general conclusion on whether a certain type of training was more effective could not be drawn due to the diversity of task characteristics and purposes, our findings show that motor imagery training has a similar effect to physical training in improving response inhibition.

In the present study, NSRT significantly increased in the MIT and MIPT groups, although no significant differences were observed in the PT group. These results conflict with the initial hypothesis for NSRT in the present study. It may be associated with a strategy of proactive inhibition. Response inhibition is not a single executive function; at least two domains have been distinguished: reactive and proactive inhibition (Mirabella, 2021). Reactive inhibition is the ability to stop a response immediately when a stop signal is presented and is quantified by measuring SSRT. Proactive inhibition is the ability to adapt the motor strategy *a priori* according to the context in which an individual is embedded (Aron, 2011; Mirabella, 2021). It could be assessed by measuring reaction times (i.e., the time to initiate a response) and the movement times (i.e., the time to execute the motor response) of no-stop trials (i.e., responding to go-signals in the SST) with those measured during the execution of the same movements in the context of a simple reaction time task (go-only trial; Mirabella et al., 2008). When a subject performs a no-stop trial, its reaction time is lengthened, and its movement time is shortened with respect to when he/she performs a go-only trial (Mirabella et al., 2008, 2013; Mancini et al., 2018). In the SST, the awareness of the possible presentation of a stop signal induces a lengthening of NSRT in order to increase the probability of suppressing the response. It allows for a better coding of the target parameters (Mirabella et al., 2008). Proactive inhibition serves as a complementary function to reactive inhibition, and is closely linked to short-, medium-, and long-term goals (Mirabella, 2021). The SST requires high cognitive demands because it consists of two tasks with opposing goals. Here, a critical aspect is that response inhibition is an explicit goal. A fast go reaction time would induce reactive inhibition with more difficulties than a slow one (Verbruggen and Logan, 2009a). For successful response inhibition, individuals in MIT and MIPT groups would employ proactive response-strategy adjustment, indicating a flexible cognitive system in changing environments. Motor imagery training can enhance the cognitive process of tasks and facilitate the planning of optimal strategies to achieve the task goals (Ladda et al., 2021). This is likely due to the fact that motor imagery training activates premotor and supplementary motor cortex, which engaged in planning and preparation (Deiber et al., 1996; Toni et al., 2001). Based on these previous studies, the MIT and MIPT groups would have developed an optimal strategy for successful response inhibition during motor imagery and adopted a proactive response strategy to extend the NSRT in order to increase the probability of stopping the response. Thus, motor imagery training could be useful for planning and adjusting response strategies with the aim of achieving successful outcomes.

## Conclusion

The present study showed that training of SST can improve response inhibition, and it was most effective when motor imagery training and physical training were combined. However, some limitations exist concerning the concluding remarks. First, it is not possible to generalize the results beyond healthy adulthood. Further studies involving the elderly or patients with neurological diseases who experience difficulties in response inhibition would allow a better understanding of this system. Second, our study only investigated the short-term effects of motor imagery training on SST. Further studies are needed to examine the long-term effects of motor imagery training on response inhibition using SST. Third, the proactive inhibition was not evaluated in the present study. In addition, the integration method, which is suitable computation approach for SSRT when proactive inhibition occurs (Verbruggen et al., 2013), could not be employed due to methodological issue. Fourth, we do not consider non-practice control group. When designing the study, we confirmed that physical training of SST improves response inhibition ability through previous studies (Kwon et al., 2011; Manuel et al., 2013). Also, many previous studies have demonstrated the effect of motor imagery training on motor performance (Robin et al., 2007; Frank et al., 2014). Although the effectiveness of each training has been proven, further studies involving a non-practice group would allow for a greater understanding of the effect of motor imagery training on response inhibition. Fifth, the neural outcomes were not considered in this study. Therefore, further studies should investigate neurological changes in regions involved in response inhibition after motor imagery combined with physical training.

## Data Availability Statement

The raw data supporting the conclusions of this article will be made available by the authors, without undue reservation.

## Ethics Statement

The studies involving human participants were reviewed and approved by the Institutional Review Board of Dankook University (DKU 2021-03-062). The patients/participants provided their written informed consent to participate in this study.

## Author Contributions

SS and JK contributed conception and design of the study. SS and SY wrote the first draft of the manuscript and conducted training of SST. SY collected SSRT and NSRT data under supervision of JK. All authors contributed to the article and approved the submitted version.

## Funding

This work was supported by the National Research Foundation of Korea (NRF) grant funded by the Korea government (MSIT) (no. 2021R1F1A1052236).

## Conflict of Interest

The authors declare that the research was conducted in the absence of any commercial or financial relationships that could be construed as a potential conflict of interest.

## Publisher’s Note

All claims expressed in this article are solely those of the authors and do not necessarily represent those of their affiliated organizations, or those of the publisher, the editors and the reviewers. Any product that may be evaluated in this article, or claim that may be made by its manufacturer, is not guaranteed or endorsed by the publisher.

## Appendix

Appendix 1 The progression of SSRT during the physical training session.

**Table:**

	Training									
	1	2	3	4	5	6	7	8	9	10
PT	231.48 ± 33.22	236.19 ± 38.63	233.35 ± 41.63	220.01 ± 47.64	222.31 ± 24.96	232.49 ± 22.30	218.43 ± 17.94	220.27 ± 22.06	213.86 ± 33.87	227.41 ± 43.29
MIT	–	–	–	–	–	–	–	–	–	–
MIPT	–	235.78 ± 23.28	–	231.59 ± 32.96	–	223.24 ± 30.32	–	227.70 ± 23.03	–	217.28 ± 19.24

PT, physical training; MIT, motor imagery training; and MIPT, motor imagery with physical training. PT group performed the physical training session twice a day, a total of 10 times for 5 days. MIT group conducted motor imagery training session twice per day, 10 times in total over the 5 days. MIPT group engaged in once motor imagery training session and once physical training sessions per day.
